# Protein-Based Oncopanel as Addition to Target Sequencing in Head and Neck Squamous Cell Carcinoma to Individualize Treatment Decisions

**DOI:** 10.3390/ijms232415835

**Published:** 2022-12-13

**Authors:** Adrian von Witzleben, Urs Müller-Richter, Katja Maurus, Stephanie Brändlein, Marie-Nicole Theodoraki, Cornelia Brunner, Simon Laban, Jochen Lennerz, Peter Möller, Thomas K. Hoffmann, Johannes Doescher, Patrick J. Schuler

**Affiliations:** 1 Department of Otorhinolaryngology and Head & Neck Surgery, Head and Neck Cancer Center of the Comprehensive Cancer Center Ulm, University Medical Center Ulm, 89075 Ulm, Germany; 2 Department of Oral and Maxillofacial Plastic Surgery, Comprehensive Cancer Center Mainfranken, University Hospital of Würzburg, 97080 Würzburg, Germany; 3 Institute of Pathology, Comprehensive Cancer Center Mainfranken, University Hospital of Würzburg, 97080 Würzburg, Germany; 4 Department of Pathology, Center for Integrated Diagnostics, Massachusetts General Hospital, Harvard Medical School, Boston, MA 02114, USA; 5 Institute of Pathology, University Medical Center Ulm, 89075 Ulm, Germany

**Keywords:** head and neck squamous cell carcinoma, immunohistochemistry, targeted therapy, oncopanel, sequencing

## Abstract

Head and neck squamous cell carcinoma (HNSCC) is a heterogeneous group of cancers and patients have limited therapy options if primary treatment fails. Therefore, additional information about the biology of the tumor is essential. Here we performed a feasibility study of concurrently applying two precision diagnostic tools in a consecutive series of HNSCC patients. We analyzed tumor samples of 31 patients using a genomic (oncomine) and a proteomic, immunohistochemical approach (oncopanel) and compared the result, also in the focus on their overlapping therapeutical targets. We found no strong correlation between the two approaches and observed a higher proportion of marker expression for the immunohistochemical panel. However, both panels show in our HNSCC cohort distinct patterns with druggable targets. The data suggest that both approaches complement one another and can be applied side-by-side to identify the best targets for the development of individual treatment options for HNSCC patients.

## 1. Introduction

Squamous cell carcinoma of the head and neck (HNSCC) is a common cancer with nearly 900,000 newly diagnosed cases per year and around 450,000 deaths per year [1]. Approximately 20–30% of HNSCCs arise in the oropharynx and a quarter of all HNSCCs are associated with human papillomavirus (HPV) while 60% of oropharyngeal squamous cell carcinomas (OPSCC) are HPV-driven [2]. Infection with high-risk HPV subtypes can lead to the development of OPSCC in individuals regardless of classic risk factors such as alcohol consumption and smoking [3].

Locoregionally advanced HNSCC are treated either surgically with subsequent risk-adapted adjuvant (chemo)radiation therapy (RT) or definitive chemoradiotherapy (CRT) [4]. The modulation of immune checkpoint molecules (ICM) and other therapeutically usable drug targets for the treatment of HNSCC is seeing increasing importance. Treatment with anti-programmed death 1 (PD1) antibodies has been established as the standard of care for platinum-naive recurrent or metastatic (R/M) HNSCC as monotherapy or in combination with cisplatin and 5-fluorouracil [5] and as monotherapy for platinum-refractory R/M HNSCC [6,7]. The combination of anti-PD1/PD-L1 treatments with conventional modalities in a curative setting is currently being investigated in various clinical studies [8,9,10]. Another targeted therapy, Cetuximab, an anti-epithelial growth factor receptor (EGFR) inhibitor, has been used for more than 10 years in R/M HNSCC as a monotherapy or in combination with platinum-based chemotherapeutic agents and taxanes [11,12,13].

Long before immunomodulation was found to be a game changer in the therapy of a subgroup of cancer patients, other therapeutically targetable cancer molecules such as tyrosine-kinase inhibitors, etc., were identified. Those potential therapeutic targets can be identified by analyzing the genetic and transcriptomic tumor landscape in head and neck tumors [4]. However, this approach can be difficult to perform in a clinical setting as it requires multiple steps of processing and is rather expensive. A cheaper and more straightforward approach could be the immunohistochemical analysis for the detection of targetable protein expression. Surface markers are the carriers of biological function and serve as target structures for many drugs. We refer to this approach as oncopanel [14,15]. Until now it was unclear whether the different approaches can detect the same targets, or if there are different signaling patterns detectable on the DNA and protein levels.

In this study, we compare our protein-based immunohistochemistry panel with targeted sequencing data assessing a variety of druggable surface markers in HNSCC patients.

## 2. Results

### 2.1. Patient Cohort

The cohort of 31 HNSCC patients contained 23 male and eight female patients. The mean age at diagnosis was 62.6 years. An oropharyngeal tumor was detected in 25 patients, of which 13 were HPV positive, and the other patients (*n* = 6) suffered from oral cavity cancer. Six patients in our cohort had a recurrence at the time of follow-up. Here we obtained matched recurrence samples from three patients with oropharyngeal cancer. One of these patients died (see individual expression profile below), and all other patients were alive at the time of follow-up. At the time of diagnosis, 54.8% (17/31) of the patients had a T2 tumor stage and 64.5% (20/31) had lymph node metastases. Most patients (28/31, 90.3%) were treated with surgery and risk-adapted radio(chemo)therapy. Primary CRT was given to three patients (9.7%) (patient data and characteristics in Table 1 and Table 2).

### 2.2. EGFR, PD-L1, and MET Are the Most Frequently Detected Targets in the Oncopanel

**Figure 1:**
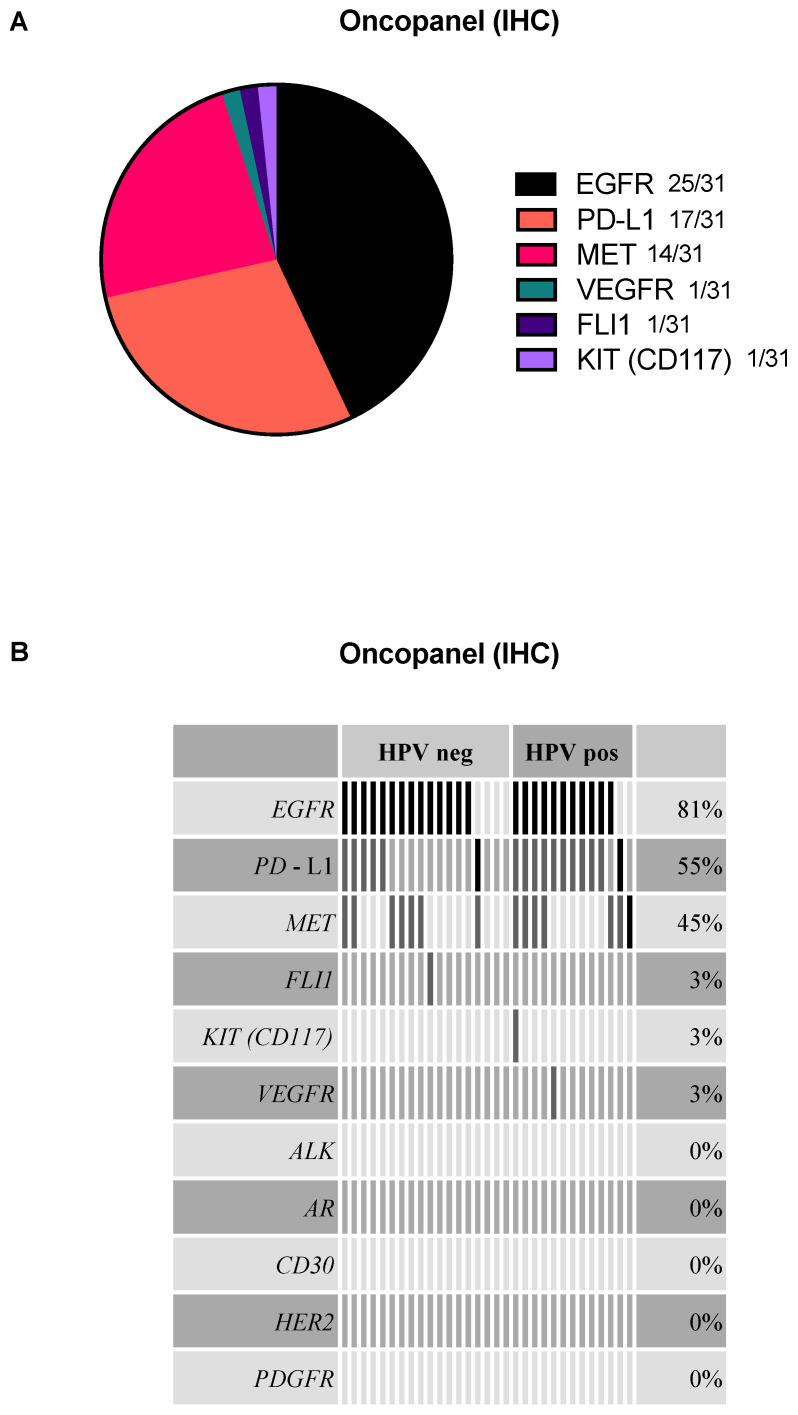
Results of the immunohistochemical approach (oncopanel) for 31 patient samples. (**A**) Pie chart displaying the distribution of marker expression. (**B**) AVAtar plot shows the markers’ distribution and their co-expression grouped by HPV status. The black bars show the first positive result per sample, while the grey bars depict a co-expression of a target in a specific sample.

Out of 31 EGFR-stained cases, 25 were positive for EGFR. The second and third most common expression was found for PD-L1 (17/31) and MET (14/31). VEGFR, FLI1, and KIT were only positive in one case each (Figure 1A). Co-expression for EGFR and PD-L1 was seen in most cases (*n* = 8, 25.8%), followed by EGFR and MET co-expression (*n* = 5, 16.1%), as well as triple co-expression of EGFR, PD-L1, and MET in five cases (16.1%) (Figure 1B). In our cohort, no expression was found for HER2, ALK, PDGFR, CD30, and AR. The T-status or nodal status had no statistically significant influence on the proteomic results.

### 2.3. Amplifications Found for Cell Cycle Genes, EGFR, and PI3-Kinase in Oncomine Targeted Sequencing

**Figure 2:**
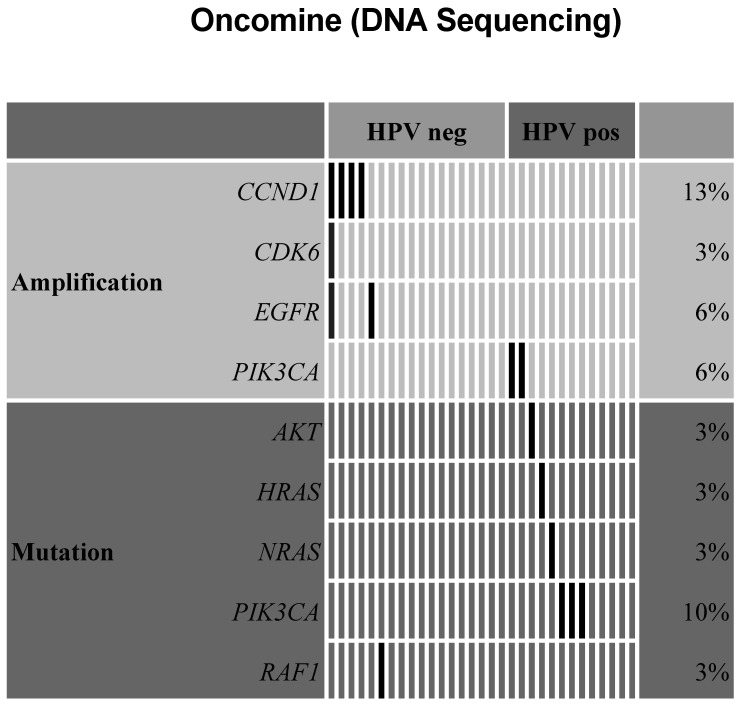
Results of the targeted mutation and amplification analysis using the oncomine panel displayed in an AVAtar graph grouped by HPV status and mutation or amplification. The black bars show the first positive result per sample, while the grey bars depict an additional finding of a target in a specific sample.

Amplification of CCND1 (encoding Cyclin D1) was found in four cases and one of those harbored an additional CDK6 amplification. An EGFR amplification was found in two cases, whereas two cases showed a PIK3CA amplification. Furthermore, we could detect oncogenic mutations within the PIK3CA gene. Mutations affecting the genes RAF1, NRAS, HRAS, AKT, FGFR1, and MTOR were found in one case each (Figure 2). The T-status or nodal status had no statistically significant influence on the genomic results.

### 2.4. Overlap of Oncopanel and Oncomine

**Figure 3:**
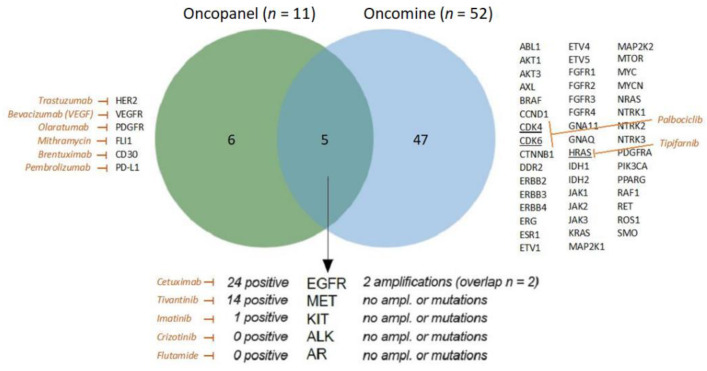
Overlapping genes/markers (*n* = 5) between the oncopanel (*n* = 11) and the oncomine panel (*n* = 52) in a Venn diagram. The results for the overlapping markers are displayed at the bottom. On the sides, a table shows the remaining genes/markers for each table. A selection of possible targets is marked with an example of targeted treatment.

The two different panels overlap in five genes/markers: EGFR, MET, ALK, KIT, and AR (Figure 3). Within the overlapping markers, only the two EGFR amplifications were matched by IHC analysis.

### 2.5. HPV-Positive and HPV-Negative HNSCC Analyzed by Oncopanel and Oncomine

A dichotomized analysis of the oncopanel immunohistochemical data showed a similar distribution of EGFR and MET expression in HPV-positive and HPV-negative HNSCC cases. In most HPV-positive cases, we detected a PD-L1 expression as expected (Figure 1B). Comparing the HPV status regarding the oncomine assay, we found CCND1, CDK6, and EGFR amplifications only in HPV-negative cases, while PIK3CA amplifications and mutations were only found in HPV-positive HNSCC (Figure 2).

### 2.6. HNSCC Recurrences Analyzed by Oncopanel and Oncomine

Matching tumor samples were available from three patients (patients #7, #10, and #16, Table 3). The comparison of the primary tumor and its relapse showed stable EGFR expression in two patients (#10 and #16) on the protein level. Interestingly, both cases displayed an acquired PD-L1 expression in the recurrent tumor whereas the primary tumor did not express PD-L1. For the genetic analysis with the Oncomine Focus Panel, amplification of CCND1 was found in the primary and recurrent tumor of #16. Patient #10 developed an amplification for CCND1 beyond progression. Analysis of case #7 did not result in any therapeutical options on the protein or DNA level.

### 2.7. Characteristics of Recurrent and Deceased Patients

**Figure 4:**
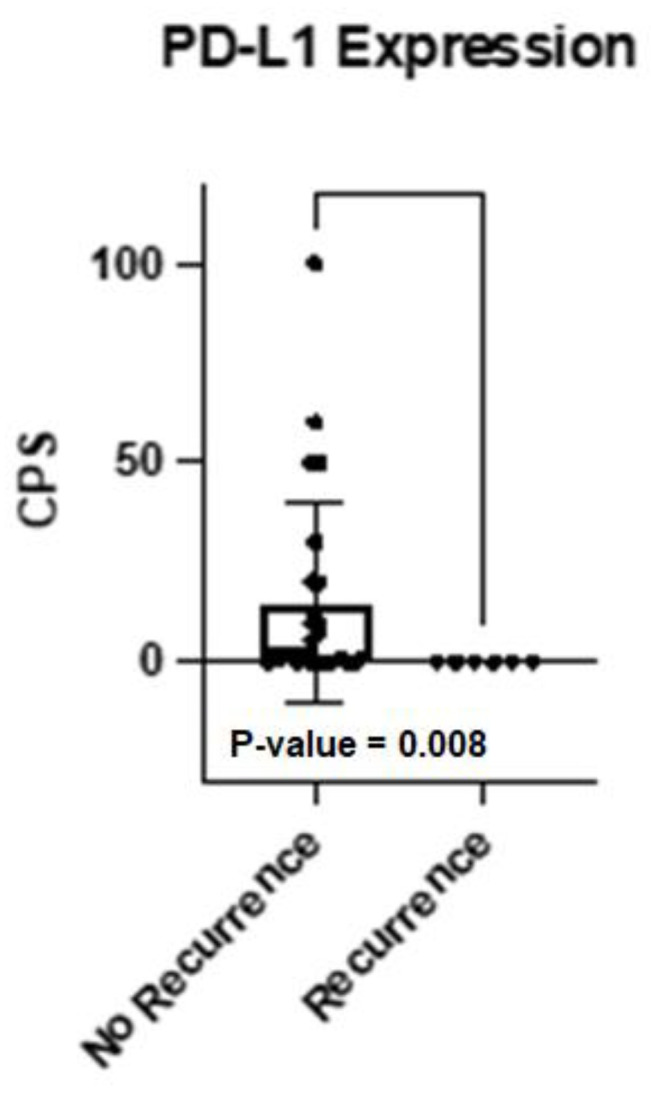
Bar graph displaying the CPS score as a percentage for all patients grouped by their recurrence status. As all tumor samples of patients with relapse in course of treatment were PD-L1-negative there is a statistically significant difference between those two groups (*p* = 0.008, Mann–Whitney test).

In our cohort, one female patient died shortly after her HNSCC recurrence was detected (#10). This case was positive for EGFR in the oncopanel, but no genetic alterations were found in the oncomine analysis. In the group of six recurrent HNSCC, no mutations were found with the oncomine panel in the primary tumor, whereas out of the 25 HNSCC cases without recurrence seven cases harbored mutations (28.0%). Amplifications were found in 2/6 recurring cases (33.0%) and 5/25 non-recurring cases (20.0%). The protein expression of the oncopanel revealed that no case with a recurrence had PD-L1 expressed in the primary tumor. Using the absolute CPS percentages, a nonparametric Mann–Whitney test revealed a statistically significant difference (*p*-value = 0.008, Figure 4). Furthermore, 5/6 cases expressed EGFR, and 3/6 cases expressed MET (Supplementary Table S2).

## 3. Discussion

In this study, we compared an immunohistochemical approach with a sequencing-based oncological panel to detect possible therapeutic targets in a cohort of 31 untreated HNSCC patients. Currently, those approaches are useful in the case of metastatic or recurrent HNSCC, where immunotherapy or other targeted therapies are often the only possible treatment options. However, these personalized targeted approaches can also become an option in curative treatment situations, if patient selection and patient benefit can be further improved.

This study shows the benefit of a straightforward proteomic approach, as the immunohistochemical analysis directly detects the targetable protein expression. Additionally, the surface markers are the carriers of biological function and therefore serve as target structures for many of the drugs. Mutations can lead to different expression patterns of RNA and subsequentially to a different protein expression. However, studies are showing that there is only a low correlation between transcriptomic and the related protein expression for genes of regulation in terms of biological processes [16]. Genome-wide correlation between mRNA expression and protein expression is poor with approximately only 40% [17].

In our study, we saw a variety of different expression patterns on the protein level and different mutation and amplification patterns. This points out the importance of inter-individual differences and illustrates the value of the personalized in-detail analysis of each patient’s tumor tissue.

In the oncopanel analysis, we observed a good proportion of tumors with co-expression of PD-L1 and EGFR. This underlines a possible treatment benefit of combining Cetuximab, an EGFR-blocking antibody, with ICM modulation. The first results of a phase 2 study, which support this promising approach, were recently published (NCT03082534) [18]. In this non-randomized, multicenter study, R/M HNSCC patients (*n* = 33) received a combination of pembrolizumab (200 mg/3 wk) and cetuximab (400 mg/m^2^ + 250 mg/m^2^/wk). After six months the overall response rate was 45% (15/33), which is more than twice as much as compared to the expected response rate with pembrolizumab monotherapy [6]. Assessment of the PD-L1 status revealed that all patients with recurrences had a negative CPS. This finding is in accordance with our previously published paper, where we could show that PD-L1 expression is a protection against the occurrence of recurrences [19].

We found an intracellular molecule CCND1, which is a protein-coding gene for Cyclin D1, to be amplified in four patients. Cyclin D1 is a protein that is involved in the cell cycle and regulates CDK4 and CDK6 as a subunit for the progression through the G1 phase of the cell cycle [20]. If this expression could be verified on the protein level as well, a possible treatment with a CDK4/CDK6 inhibitor such as Palbociclib or Abemaciclib could be attempted. Therefore, the next upgrade for the oncopanel would be an additional IHC marker with Cyclin D1, maybe also with CDK4 and CDK6. A phase I study evaluated Palbociclib in R/M HNSCC safe with tumor responses, and in vitro experiments combined with an mTOR inhibitor show promising anti-tumor effects (Figure 3) [21,22].

With the oncomine assay, we detected an activating mutation of HRAS (Figure 2 and Figure 3), which can be targeted using Tipifarnib a farnesyltransferase-inhibitor. This targeted therapy showed promising results in a phase II clinical trial of R/M HNSCC with a median progression-free survival of 5.6 months on tipifarnib versus 3.6 months on the last prior therapy (NCT02383927) [23].

Interestingly, we could find a different genomic and proteomic pattern in HPV-negative vs. -positive HNSCC. For example, cell cycle aberrations were only found in HPV-negative cases, while PIK3CA amplifications and mutation were solely found in HPV-positive cases. This underlines the importance of stratifying HNSCC in HPV-positive and HPV-negative also in the clinical decision finding.

The two panels were used as available, clinically proven panels containing each therapeutically targetable markers and genes. Therefore, the targets are not overlapping completely, however, they complement each other. The combination of both approaches is in our opinion necessary, as well as complementary, as not every amplification leads to an (over-)expression of protein, and other important regulatory mechanisms are involved. However, it would be interesting if following studies could assess all genes of interest on genomic, transcriptomic, and proteomic levels and could include analyses such as methylome or miRNA screens. Subsequently, a sound statement could be made about how DNA, RNA, and protein expression correlate, as well as how other gene regulatory mechanisms are involved, and how it might differ specifically for each gene of interest. However, this is rather expensive and labor intensive and is probably not feasible in the clinical routine. One key limitation of all DNA-based genotyping assays is that the functional consequences are usually “predicted” rather than directly assessed. Mutation-specific antibodies (REF) or immunohistochemistry in general (PD-1/PD-L1) can overcome this limitation and can directly visualize the pharmacologic targets in situ. Proteins are the carriers of biological function; our approach to combining DNA and protein assessment acknowledges this biological paradigm. Another limitation might be the small number of cases, but, on the other hand, we carefully selected a rather homogeneous cohort for the best comparison within cases.

An additional interesting point to mention is the similar costs to perform the assays. The immunohistochemical oncopanel costs EUR 350 related to the German fee schedule, while the material for the oncomine panel costs for one sample also around EUR 300. Here is to add, that the hands-on time for the sequencing is more labor-intensive than the oncopanel, and the analysis is more complex and needs additional equipment to perform those tasks, such as sequencers, servers, and computing power.

In summary, we conclude that the combination of the oncopanel and the oncomine assay brought up several targetable genetic and expression changes in individual patients, which can lead to the repurposing of available drugs and opens a variety of treatment options for those patients.

## 4. Materials and Methods

### 4.1. Study Design and Patient Cohort

Following a previously published study with heavily pre-treated HNSCC patients [15], we now established a prospective cohort of 31 previously untreated HNSCC patients at the time of their primary surgical treatment and used those in this explorative prospective study (Table 1). It was IRB approved (ethics 90/15) and patients signed informed consent before biopsy and any kind of treatment. The samples were collected either at the time of tumor biopsy or at the time of major surgery, and the samples were referred to both immunohistochemistry staining and targeted DNA sequencing (Oncomine Focus Assay, an NGS tumor-specific panel, ThermoFisher, Waltham, MA, USA) (Table 2). Further, for three of the patients a specimen for subsequent analysis at the time of recurrence was available.

### 4.2. Oncopanel

The oncopanel in this study assessed the surface expression of 11 proteins (EGFR, MET, HER2, ALK, VEGFR, PDGFR, FLI1, KIT (CD117), CD30, AR, PD-L1) as previously published (15). The staining was performed on the Dako Omnis platform (Dako, Glostrup, Denmark) as previously published [14,15]. Due to the very low prevalence of BRAF V600E in non-melanocytic head and neck carcinoma, we modified the oncopanel and excluded this marker. We also excluded PD-1 as a marker because PD-L1 expression has been established as a predictor of response to anti-PD-1 therapy and made the routine assessment of PD-1 expression redundant [6]. Briefly, we used formalin-fixed paraffin-embedded tissues and antibody details are provided in the Supplementary Table S1. Cases were considered positive if more than 10% of tumor cells showed immunopositivity in the appropriate cellular compartment. PD-L1 expression was scored according to the combined positive score (CPS) and cases with a CPS ≥ 1 were considered positive.

### 4.3. DNA Extraction

Archival formalin-fixed paraffin-embedded (FFPE) tissue specimens of squamous cell carcinoma patients were microdissected and genomic DNA was extracted with the Maxwell RSC Blood DNA Kit after a pre-treatment with a THG1-Thioglycerol/incubation buffer mix for 10 min at 80 °C and subsequent incubation with proteinase K at 65 °C overnight (Promega, Walldorf, Germany). DNA from cryo-preserved material was extracted using the DNeasy Blood and Tissue Kit (Qiagen, Hilden, Germany). DNA was extracted from fresh frozen samples (*n* = 16) and 15 FFPE samples.

### 4.4. Next Generation Sequencing (NGS)

To determine the concentration of amplifiable genomic DNA, we performed qPCR of the 34 extracted DNA samples (TaqMan RNase P Detection Reagents Kit, ThermoFisher, Waltham, MA, USA). Libraries were prepared with a multiplex PCR approach using the Oncomine Focus Assay and the Ion AmpliSeq Library Kit 2.0 (both ThermoFisher, Waltham, MA, USA) for 52 genomic targets (Figure 3). Libraries were templated and enriched with the Ion OneTouch 2 and the Ion OneTouch ES automated systems (both ThermoFisher, Waltham, MA, USA). Sequencing was performed using semiconductor sequencing technology (Ion GeneStudio S5, ThermoFisher, Waltham, MA, USA; sequencing quality is shown in Supplementary Table S3). Bam-files were generated by Torrent Suite software, version 5.10, and variant annotation was performed by the Ion Reporter software with default parameters for the Oncomine Focus Assay, version 5.10 (ThermoFisher, Waltham, MA, USA).

### 4.5. Data Analysis and Statistics

Data were analyzed and graphed using Microsoft Excel for Mac (version 16.54) and GraphPad Prism (version 9.2.0). The upset plot was generated in R (version 4.0.3) with RStudio (version 1.2.5033) and the ComplexHeatmap package using the UpSet function [24]. The analysis and visualization of the oncopanel and oncomine data were performed in the standalone software AVAtar (https://github.com/sysbio-bioinf/avatar, accessed on 10 December 2022) [25]. The Venn diagram was generated using the online javascript jvenn [26].

## 5. Conclusions

Untreated HNSCC patients display a series of targetable alterations in protein expression and DNA levels, which can be detected by immunohistochemistry and DNA sequencing. Both relatively cost-effective approaches complement one another and can be applied side-by-side to identify the best treatment option for HNSCC patients. Currently, those approaches are used in metastatic or recurrent HNSCC, where immunotherapy or other targeted therapies are often the only possible treatment options. However, these personalized targeted approaches can also become an option in curative treatment situations if patient selection and patient benefit can be further improved.

## Figures and Tables

**Table 1:** Tabular patient characteristics with their ID, gender, age, life status, recurrence status, HPV status, localization, TNM Status, and treatment.

PatID	Gen-der	Age	Life Status	OS	Recurrence	PFS	HPV Status	Localization	T-Status	N-Status	M-Status	Therapy
1	m	52	alive	35	no		negative	Oropharynx	3	1	0	SRG+RT
2	m	47	alive	35	no		negative	Oropharynx	3	1	0	SRG+RT
3	m	65	alive	35	no		positive	Oropharynx	1	1	0	SRG+CRT
4	m	61	alive	26	no		positive	Oropharynx	2	2b	0	SRG+CRT
5	m	71	alive	32	yes	16	positive	Oropharynx	4	2c	0	CRT
6	m	50	alive	35	no		negative	Oropharynx	2	0	0	SRG
7	f	59	alive	33	yes	34	negative	Oropharynx	2	0	0	SRG
8	m	69	alive	34	no		negative	Oropharynx	2	3b	0	SRG+CRT
9	m	71	alive	22	no		negative	Oropharynx	2	2b	0	SRG+RT
10	f	54	dead	10	yes	8	negative	Oropharynx	2	0	0	SRG+RT
11	m	68	alive	32	no		positive	Oropharynx	2	2c	0	SRG+RT
12	m	64	alive	29	no		positive	Oropharynx	2	0	0	SRG
13	m	54	alive	29	no		negative	Oropharynx	4	2c	0	SRG+CRT
14	m	58	alive	26	yes	23	negative	Oropharynx	3	0	0	CRT
15	m	56	alive	28	no		positive	Oropharynx	2	1	0	SRG+CRT
16	f	56	alive	27	yes	5	negative	Oropharynx	4	2a	0	SRG
17	f	38	alive	25	no		na	Oral Cavity	2	0	0	SRG+RT
18	m	69	alive	19	no		na	Oral Cavity	3	1	0	SRG
19	f	66	alive	21	no		na	Oral Cavity	3	1	0	SRG+RT
20	f	69	alive	19	no		na	Oral Cavity	2	0	0	SRG
21	m	64	alive	15	no		positive	Oropharynx	3	2c	0	CRT
22	m	64	alive	14	no		positive	Oropharynx	2	1	0	SRG+RT
23	m	83	alive	14	no		positive	Oropharynx	2	1	0	SRG+RT
24	m	76	alive	16	no		positive	Oropharynx	2	0	0	SRG+RT
25	m	68	alive	13	no		negative	Oropharynx	2	2b	0	SRG
26	f	64	alive	1	no		positive	Oropharynx	4	2a	0	SRG+CRT
27	f	57	alive	12	no		positive	Oropharynx	2	0	0	SRG
28	m	76	alive	13	yes	6	negative	Oropharynx	4	2b	0	SRG+RT
29	m	67	alive	13	no		positive	Oropharynx	1	2a	0	SRG+RT
30	m	63	alive	6	no		na	Oral Cavity	1	0	0	SRG+RT
31	m	64	alive	6	no		na	Oral Cavity	2	0	0	SRG

m = male; f = female; SRG = surgery; RT = radiotherapy; CRT = chemoradiotherapy; OS = overall survival in months; PFS = progression-free survival in months.

**Table 2:** Summarized patient characteristics.

		*n*	%
Primary Site	Oropharynx	25	81%
	Oral Cavity	6	19%
	Total	31	
Sex	Male	23	74%
	Female	8	26%
Age	mean (range)	62.6 (37.9–83.0)	
Tumor Status	T1	3	10%
	T2	17	55%
	T3	6	19%
	T4	5	16%
Nodal Status	N-	11	35%
	N+	20	65%
Distant Metastases	M0	31	100%
HPV-status (DNA)	negative	12	
	positive	13	
	missing (not OPSCC)	6	
Treatment	CRT only	3 (2 *)	
	Surgery only	9	
	Surgery+RT	6	
	Surgery+CRT	13	

HPV = human papillomavirus, RT = radiotherapy, CRT = chemoradiotherapy, * with Pembrolizumab.

**Table 3:** Tabular comparison of the primary and recurrent tumor tissue for both panels. Patient IDs are displayed as defined in Table 1.

	Primary	Recurrence
	Oncopanel (IHC)	
**Patient 7**	*no target*	*no target*
**Patient 10**	EGFR+	EGFR+/PD-L1
**Patient 16**	EGFR+	EGFR+/PD-L1
	Oncomine (amplification)	
**Patient 7**	*no target*	*no target*
**Patient 10**	*no target*	CCND1
**Patient 16**	CCND1	CCND1

## Data Availability

The datasets were submitted to the European Genome-phenome Archive and will be available soon at https://www.ebi.ac.uk/ega/datasets (accession number: EGAS00001006296).

## Supplementary Materials

The following supporting information can be downloaded at: https://www.mdpi.com/article/10.3390/ijms232415835/s1.
