# Supplementary material for: Protein-Based Oncopanel as Addition to Target Sequencing in Head and Neck Squamous Cell Carcinoma to Individualize Treatment Decisions
Source: Int J Mol Sci. 2022 Dec 13;23(24):15835. doi: 10.3390/ijms232415835 (PMC9779552; doi:10.3390/ijms232415835)
Supplement: Supplementary file 1 [file ijms-23-15835-s001.zip › suppl. table 1.pdf]

Table S1

Name	Host	Clone	Dilution	Staining pattern
ALK	Ms, m	ALK1, M7195 (Dako)	1:100	cytoplasmic
AR	Ms, m	AR441 (Dako)	1:200	nucleus
EGF-R	Ms, m	2.1E1, MSK014-05 (Zytomed)	1:100	membranous
FLI-1	Rb, p	N/A, RB9295 (Medac)	1:100	nuclear
HER2	Rb, p	N/A, A0485 (Dako)	1:500	membranous
KIT	Rb, p	N/A, A4502 (Dako)	1:200	cytoplasmic
MET	Rb, m	Met D1C2, 8198 (Cell Signaling)	1:100	cytoplasmic/ membranous
PD-L1	Rb, m	E1L3N (Cell Signaling)	1:400	membranous
PDGF-R	Rb, m	D13C6, 5241S (Cell Signaling)	1:25	cytoplasmic
TNF-R8 (CD30)	Ms, m	Ber-H2 (Cell Marque)	1:25	membranous
VEGF-R	Ms, p	N/A, DP067 (Acris)	1:200	cytoplasmic

*Acris, Herford, Germany; Dako, Braunschweig, Germany; Cell Marque, Rocklin, USA; Cell Signaling, Frankfurt, Germany; Medac, Wedel, Germany; Zytomed Systems, Berlin, Germany
