# Supplementary material for: Protein-Based Oncopanel as Addition to Target Sequencing in Head and Neck Squamous Cell Carcinoma to Individualize Treatment Decisions
Source: Int J Mol Sci. 2022 Dec 13;23(24):15835. doi: 10.3390/ijms232415835 (PMC9779552; doi:10.3390/ijms232415835)
Supplement: Supplementary file 1 [file ijms-23-15835-s001.zip › Supplement Table Legends.pdf]

Supplementary Tables:

**Suppl. Table 1:** Antibody specifications. Ms: mouse; Rb: rabbit; m: monoclonal; p: polyclonal.

**Suppl. Table 2:** Tabular overview of the genomic and proteomic expression of the primary tumors grouped by their recurrence status.

**Suppl. Table 3:** Tabular overview of the sequencing data, the quality of each sample and the amplification and mutation data.
